# Elemental iron protects gut microbiota against oxygen-induced dysbiosis

**DOI:** 10.1371/journal.pone.0298592

**Published:** 2024-02-27

**Authors:** Ievgeniia Ostrov, Yongjia Gong, Joshua B. Zuk, Purni C. K. Wickramasinghe, Irina Tmenova, Diana E. Roopchand, Liping Zhao, Ilya Raskin

**Affiliations:** 1 Department of Plant Biology, School of Environmental and Biological Sciences, Rutgers University, New Brunswick, New Jersey, United States of America; 2 Department of Food Science, School of Environmental and Biological Sciences, Rutgers University, New Brunswick, New Jersey, United States of America; 3 Department of Biochemistry and Microbiology, School of Environmental and Biological Sciences, Rutgers University, New Brunswick, New Jersey, United States of America; Wageningen Universiteit, NETHERLANDS

## Abstract

Gut dysbiosis induced by oxygen and reactive oxygen species may be related to the development of inflammation, resulting in metabolic syndrome and associated—conditions in the gut. Here we show that elemental iron can serve as an antioxidant and reverse the oxygen-induced dysbiosis. Fecal samples from three healthy donors were fermented with elemental iron and/or oxygen. 16S rRNA analysis revealed that elemental iron reversed the oxygen-induced disruption of Shannon index diversity of the gut microbiota.The bacteria lacking enzymatic antioxidant systems also increased after iron treatment. Inter-individual differences, which corresponded to iron oxidation patterns, were observed for the tested donors. Gut bacteria responding to oxygen and iron treatments were identified as guilds, among which, *Escherichia-Shigella* was promoted by oxygen and depressed by elemental iron, while changes in bacteria such as *Bifidobacterium*, *Blautia*, *Eubacterium*, *Ruminococcaceae*, *Flavonifractor*, *Oscillibacter*, and *Lachnospiraceae* were reversed by elemental iron after oxygen treatment. Short-chain fatty acid production was inhibited by oxygen and this effect was partially reversed by elemental iron. These results suggested that elemental iron can regulate the oxygen/ROS state and protect the gut microbiota from oxidative stress.

## Introduction

Metabolic syndrome (MetS) is a growing health crisis, requiring effective diagnostics, therapies, and prevention strategies [1]. MetS is characterized by a clustering of at least three of the five following cardiovascular disease (CVD) risk factors, obesity, hyperlipidemia, hypertension, hyperglycemia, and low serum high-density lipoprotein [2, 3]. This health condition has been shown to lead to a prothrombotic and a proinflammatory state and increase risk for development of CVD, diabetes, nonalcoholic fatty liver disease, liver cirrhosis in chronic hepatitis B, and liver cancer [3–7].

Studies conducted with both human and animal models suggest that gut microbiota play an integral role in the pathogenesis of MetS [8]. Gut microbiota consist of a wide variety of microbes inhabiting the colon and correlate with human health and diseases. Oxygen and its derivatives play an important role in the oxidative stress-induced dysbiosis of gut microbiota, which further contributes to the progression of MetS and other diseases such as inflammatory bowel diseases (IBD). Oxidative stress can strongly decrease microbial diversity in the gut through inhibiting the growth of obligate anaerobes that are susceptible to oxygen toxicity. Presence of oxygen and reactive oxygen species (ROS) can also promote the growth of facultative anaerobic bacterial taxa, which include human pathogens [9]. Previous studies have shown a decrease in obligate anaerobic bacteria—especially *Faecalibacterium prausnitzii*, and an increase in facultative anaerobic bacteria—such as *Escherichia coli* (belonging to the family *Enterobacteriaceae*) that occurred in the stool samples collected from IBD patients when compared to healthy individuals [2–4]. Moreover, ROS could attack the host macromolecules leading to cellular dysfunction including loss of energy metabolism, genetic mutations, and overall decreased biological activity, immune activation and inflammation. Increased biomarkers of oxidative stress and decreased antioxidant defenses have been measured in blood of patients with MetS suggesting overproduction of ROS [10, 11]. In addition, nutritional stress such as that caused by a high fat diet (HFD) and/or high carbohydrate diet and continuous hypernutrition also promotes oxidative stress, leading to increased lipid peroxidation products, protein carbonylation, decreased antioxidant system, and reduced glutathione levels [12–14]. It was also shown that consumption of HFD leads to the alteration of gut microbiota and the production of lipopolysaccharide (LPS) that can enter circulation and reinforce the generation of ROS and inflammation [5, 12, 15]. ROS coupled with inflammation and microbial dysbiosis, lead to impaired energy and glucose metabolism, insulin resistance and visceral adiposity, which were featured in MetS patients [6, 16, 17]. Previous studies have shown that antioxidants, such as oligomeric polyphenols, can neutralize the oxygen and ROS in the gut to promote growth of beneficial obligate anaerobic bacteria and attenuate symptoms of MetS and IBD [7–9].

Iron is a necessary nutrient for living organisms. In humans, iron is involved in oxygen transport, enzyme catalysis, and regulation of the immune system [18, 19]. Iron is typically supplemented to treat anemia in a soluble and bioavailable ferrous salt form. Elemental iron (Fe^0^) is iron in its fully reduced form with an oxidation state of zero. Fe^0^ acts as an effective electron donor that reduces ROS and scavenges oxygen. For this reason, it is often used as a commercial oxygen scavenger [20].

Recently, we found that Fe^0^ confered protection against GI-localized oxidative stress by scavenging oxygen in the GI lumen of HFD-fed mice [21]. Fe^0^ acted as a non-invasive sensor of intestinal ROS as well as a non-bioavailable antioxidant that acts entirely within the GI tract. Treatment of HFD-fed mice with Fe^0^ improved glucose tolerance as well as increased potentially beneficial obligate anaerobic bacteria, including *Akkermansia muciniphila*, within the mouse gut. However, the influence of Fe^0^ supplementation on the human gut microbiome and metabolic health markers remains unknown.

We hypothesized that elemental iron facilitates survival of beneficial anaerobic bacteria by neutralizing excess oxygen and ROS, which may initiate the events that lead to the attenuation of MetS and IBD. To test our hypothesis, we used an *in vitro* model system for evaluating the response of human gut microbiota to Fe^0^ administration in microaerophilic conditions, coupled with a method for evaluating the oxygen-scavenging ability of Fe^0^.

## Materials and methods

### Materials

325 mesh reduced iron powder was purchased from Beantown Chemical (Hudson, NH, USA). Basal culture medium (BCM, eutrophic) were prepared with peptone at 2g/L, yeast extract at 2g/L, MgSO4 7H2O at 10 mg/L, KH2PO4 at 40 mg/L, NaHCO3 at 2 g/L, NaCl at 0.1 g/L, KH2PO4 at 40 mg/L, bile salts at 0.5 g/L, CaCl2.6H2O at 10 mg/L, Tween 80 at 2 mL/L, vitamin K at 10 μL/L, and hemin at 5 mg/L. PBS tablets is reconstituted to 0.01 M phosphate buffer, 0.0027 M potassium chloride and 0.137 M sodium chloride. Maximum recovery diluent contained PBS tablets at 5 tablets/L, peptone at 1g/L, and resazurin at 1mg/L. All the chemicals were purchased from Sigma-Aldrich (St Louis, MO, USA).

### In vitro batch fermentation

Elemental iron was introduced to the culture system by weighing it into empty 20mL Hungate tubes (12.5mg/tube) in the iron-containing groups and capped and sealed Hungate tubes were purged with scrubbed N_2_ (Airgas, NJ, USA) at 15 psi for 45 sec to expel oxygen, while empty tubes (not containing iron) were purged for the control and oxygen groups (see Table 1). Purged Hungate tubes with elemental iron were autoclaved at 121°C for 15 min, then placed into an anaerobic chamber (90% N2, 5% CO2, 5% H2 gas mixture; Bactron 300, Sheldon Manufacturing, Cornelius, OR, USA). To obtain the sterile air, 10mL serum bottles were capped and sealed in the ambient environment and autoclaved at 121°C for 15 min and transferred into the anaerobic chamber.

**Table 1:** Treatment groups for *in vitro* fecal fermentation.

Treatment group	Treatment details
control	contained no oxygen + no iron
iron	contained 2.5g/L iron + no oxygen
oxygen	contained 5% oxygen + no iron
oxygen-iron	contained 5% oxygen + 2.5g/L iron

Basal culture medium (BCM) was used to simulate high fat-, high protein-diet and maximum recovery diluent (MRD) was used to prepare the fecal slurry according to the previous protocol [22]. BCM was modified by mucin (From Porcine Stomach, Type III, Himedia) supplementation at 2.5 g/l and removing reducing agent cysteine hydrochloride. MRD medium was also modified by removing cysteine hydrochloride [23–25]. The growth media were purged with scrubbed N_2_ through a fine diffusing stone at 15 psi for 4 hours to allow deoxygenation of the media. Afterwards, the media were autoclaved at 121°C for 20 min and transferred into the anaerobic chamber.

Three healthy individuals (male, age 29; female, age 40; female, age 60) were recruited under the IRB #2019000885. Written consents were provided. The recruitment period began on July 9, 2019 and ended on April 22, 2021.

The human donors’ stool samples were used for *in vitro* fermentation in BCM. Details on the four treatment groups are provided in Table 1.

For *in vitro* fermentation fresh fecal samples from each donor were collected in the morning of the experiment. The samples were transported to the anaerobic chamber within 2 hours. After homogenizing the fecal sample with MRD medium at ratio of 1:3 (w/v), fecal slurry was filtrated with 4 layers of cheese cloth to remove the residual particles. 1 portion of filtrated fecal slurry was mixed with 24 portions of BCM medium to get 1% fecal inoculum. 5mL of the fecal inoculum was injected into each sealed Hungate tube. 3.57mL of headspace gas was withdrawn from the Hungate tubes and the same amount of sterile air was further injected into the Hungate tubes in the oxygen-containing groups. Three replicates were used for each group and fermented for 12 hours at 37°C on a horizontal shaker (120rpm). Samples were collected at 0 hour and 12 hours of fermentation and centrifuged to get supernatant and pellets to store at -80°C until iron oxidation measurement, SCFA measurement, and 16S rRNA sequencing.

### Modified ferrozine assay

A modified ferrozine assay was carried out to determine relative iron oxidation as described previously [21]. Fecal pellets containing iron powder (a mixture of Fe^0^ and Fe^2+/3+^) were collected from 3 samples each containing either 0% or 5% O_2_ at each time point for each of the 3 donor fecal samples. 1 mL of 0.5 M HCL was added to the tube containing the fecal pellet and vortexed A small piece of weigh paper was placed on top of the homogenized fecal sample and a neodymium magnet was placed on top of the weigh paper. The magnet and paper were pressed against the top of the tube and the tube was inverted three times to collect the iron powder from the sample. The paper and magnet were then removed from the tube with the collected iron powder. The iron powder was washed with 1 ml Millipore water then collected in a 5 mL centrifuge tube by removing the magnet from the weigh paper and rinsing the iron powder into the tube with 1 mL 5% hydrogen peroxide to oxidize it to Fe_2_O_3_. The sample was vortexed while periodically opening the tube to relieve pressure. 10 μL of the homogenized fecal sample with iron powder removed was added to a tube containing 990 μL 0.5 M HCl and the tube was vortexed.10 μL from the tube containing the oxidized iron solution was added to another tube with 990 μL 0.5 M HCl and vortexed. 10 μL of each sample was added to a 96-well plate in triplicate. 10 μL of each concentration of an FeCl_3_ standard curve was also added in triplicate. The volume of each well was brought to 100 μL with 90 μL of total Fe buffer (0.2 mg/mL Ferrozine, 11.9 mg/mL HEPES, 10 mg/mL hydroxylamine HCl, adjusted to pH 7) and the plate was incubated for 1 h at 30°C. Absorbance at 562 nm was measured using a Biotek Synergy HT Multi-Detection Plate Reader (Winooski, VT).

### Short chain fatty acid (SCFA) extraction and analysis

SCFA extraction and analysis was done as described previouslyby the New Jersey Institute for Food, Nutrition, and Health (IFNH) Lipidomics Core and Creative Proteomics [26]. Samples were homogenized for 1 min at 22.5 rpm in 30 mM hydrochloric acid with 10mM methyl-heptanoate internal standard. Water was added to reach a final volume of 400 μl. 250 μl of methyl tert-butyl ether was added to each sample and vortexed, then held at 4°C for 5 min and vortexed again. Solvent layers were separated by centrifuging at 500 x g for 3 minutes at ambient temperature. The upper layer (methyl tert-butyl ether) was then transferred to a vial for GC-MS analysis. GC-MS analysis was performed on an Agilent 7890B GC-5977B MS detector (Agilent, Santa Clara, CA). DB-WAX column (30 m × 0.25 mm × 0.25 μm GC column; no. 122–7032; Agilent) was used with helium carrier gas. Data were processed with Masshunter Quantitative analysis software version B.07.06.2704. Data are presented as ng/μL of SCFAs.

### DNA extraction and sequencing

DNA was extracted with Protocol Q as previously described [27]. The hypervariable region V4 of 16S rRNA gene was amplified with fusion primer 515F and 806R [28, 29]. 515 Forward primer sequence was GGACTACNVGGGTWTCTAAT. The adapter sequence was TTACCGCGGCKGCTGRCAC. The forward primers were universal, and the reverse primers were ligated to adapters and unique barcodes (ThermoFisher Scientific, Waltham, MA, USA). Polymerase chain reaction (PCR) was set up with 10μl of the Platinum^™^ SuperFi^™^ DNA Polymerase (ThermoFisher Scientific, Waltham, MA, USA), 1μl of the forward primer and 1μl of the reverse primer each at a final concentration of 1uM, 20ng of DNA template and 6 μl of nuclease-free water to reach 20 μl of the final PCR reaction mixture. A mock community with genomic DNA (BEI Resources, Manassas, VA, USA) was included in the sequencing run to test the consistency between each run. DNA templates were amplified with the Mastercycler nexus GX2 (Eppendorf, Hauppauge, NY) using the following program: 98°C x 30 sec, (98°C x 8 sec + 59.6°C x 10 sec + 72°C x 10 sec) for 30 cycles and 72°C x 5 min. PCR products were purified with the AMPure XP beads (Beckman Coulter, Indianapolis, IN, USA) in 1:1.5 ratio of sample to beads to remove primer dimers. Purified PCR products were quantified by Qubit 4 (ThermoFisher Scientific, Waltham, MA, USA) and diluted to 30pM and pooled together into a library. 25μl of the library was used to template an Ion chip and the chip was sequenced by Ion GeneStudio S5 (ThermoFisher Scientific) following the manufacturer’s protocol.

### Bioinformatic and statistical analysis

A total of 72 fermented samples (4 groups, 4 timepoints, 5 replicates, 3 donors) were collected for 16S rRNA gene V4 sequencing. Primers were trimmed using cutadapt in QIIME2 software [30, 31]. After denoising by the dada2 denoise-single command with parameters—p-trim-left 0 –—trunc-len 215.Amplicon sequence variants (ASVs) were obtained and spurious ASVs were removed by abundance filtering [32, 33]. ASVs with its lower bound of confidence interval 99% is below zero are considered unrelizable and were removed. Q2-feature-classifier plugin (26) based on the SILVAdatabase (release 132) was performed to assign taxonomy of the ASVs in QIIME 2 [34]. The sequencing data were rarified to 16,000 reads/sample based on rarefaction curve for following analysis. ASVs shared by more than 30% of all samples were selected as prevalent ASVs. Those prevalent ASVs were grouped with the Ward clustering algorithm based on the repeated measurement correlation [35]. Permutational multivariate analysis of variance (PERMANOVA; 9999 permutations with a p<0.001 cutoff) were applied to determine the significant difference between two clades and assign the clades with no significant difference to same CAGs as co-abundance groups. Based on Bray Curtis distance, covariate Adjusted Principal Coordinates Analysis (aPCoA) was performed with adjusting donor effect at CAGs and ASVs profiles level using R VEGAN package version 2.5–6 and R aPCoA package version 1.2 [36]. Statistical analysis of alpha diversity was performed using Prism 9 (GraphPad Software, San Diego, CA). Two-way ANOVA along with Tukey’s and Šidák’s multiple comparison tests were used to examine the different treatments at the same time and the same treatment at different times. Procrustes analysis was performed to visualize and analyze the significance of the similarity of the shapes between aPCoA plots. MaAsLin2 were applied to identify the correlations and significance between CAGs abundance and treatments with control group as reference [37]. CAG network was visualized using Cytoscape (Version 3.9.0). Figures were generated with R package ggplot2 and pheatmapfig. Functional prediction of ASVs was performed using PICRUSt2 [37]. Donor stratified PERMANOVA was performed to compare the significance of the gut microbial community dissimilarity between groups. Sequencing data is available at NCBI BioProject ID PRJNA986515.

## Results

### Elemental iron scavenged oxygen in the *in vitro* fermentation system

**Fig 1:**
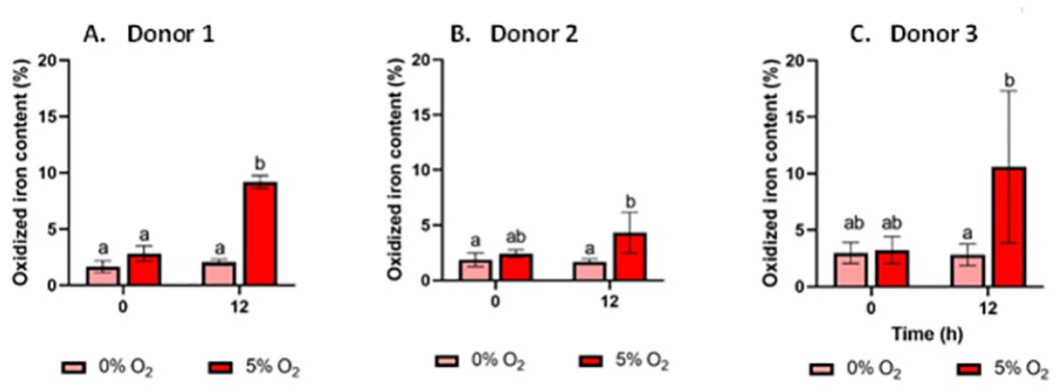
Quantification of iron oxidation by modified ferrozine assay analysis. A) B) C)Percent oxidized iron content was measured at 0 and 12 hours of fermentation in samples from donor 1, 2, and 3 with either 0% or 5% O_2_ (n = 3). Data is shown as mean ± SEM and was analyzed with two-way ANOVA and Tukey’s multiple comparisons test. Different letter subscripts indicate significant difference between groups (*p*< 0.05). a and b represent statistically different treatments, while ab is not statistically different from both a and b. Treatments having the same letter subscript are not significantly different from each other.

To determine the extent of elemental iron oxidation in the fecal fermentation system, a modified ferrozine assay was used to measure the concentration of Fe^2+/3+^ compared to Fe^0^ at baseline and 12h in iron and oxygen-iron groups for all three donors (Fig 1A–1C). No significant difference was observed between the baseline samples of all three donors. Iron oxidation patterns were measured for 0, 2, 12, 24 and 48h for one of the donors (donor 1). There was no statistically significant difference in oxidized iron content in the samples exposed to oxygen for 12h compared to 2h and 24h (see S5 Data). Therefore, the analysis was performed for samples obtained at 0h and 12h of fermentation. After 12 hours of fermentation, iron groups without oxygen showed no significant difference from baseline samples, indicating that iron was not oxidized by fecal bacteria in the anoxic environment. Oxidized iron contents were significantly higher in oxygen-iron groups than iron groups at 12 hours in all three donors, suggesting that iron was oxidized because of the addition of oxygen. Of note, average oxidized iron content in donor 2 was 4% while approximately 10% in donor 1 and donor 3 at 12 hours. Oxidized iron content was significantly lower in donor 2 compared with donor 3 (p = 0.0016), and showed a trend for significance with donor 1 (p = 0.0504), which may indicate that the oxygen-scavenging effect of elemental iron in donor 2 was lower than in donor 1 and 3.

### Elemental iron reversed the oxygen-induced dysbiosis of the gut microbiota with significant inter-individual variations

**Fig 2:**
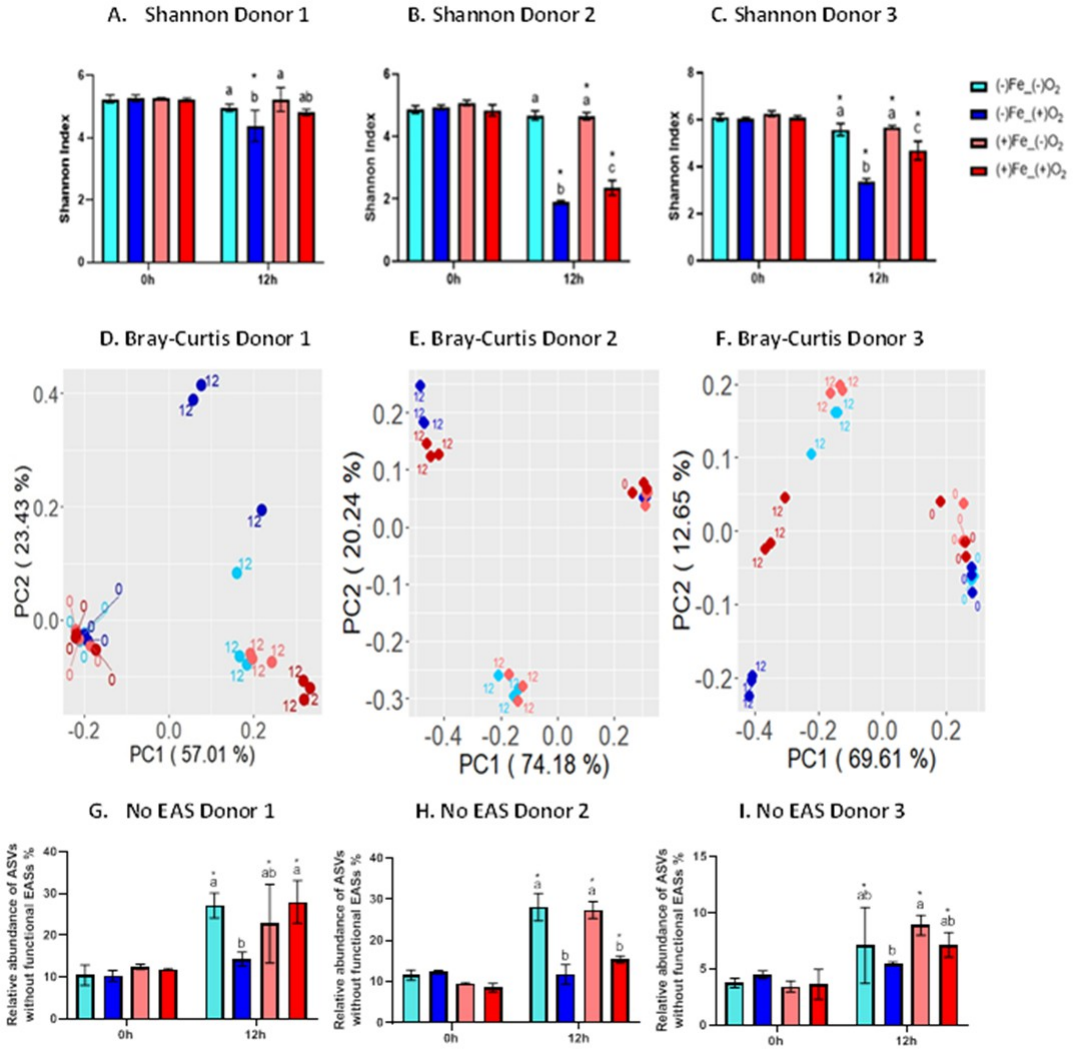
Elemental iron partially reversed the oxygen-induced microbiota alteration after 12 hours of *in vitro* fermentation. A) B) C) Alpha diversity of gut microbiota measured by Shannon index at baseline and 12 hours from Donor 1, 2, and 3. Data shown as mean ± SEM and compared with two-way ANOVA and Tukey’s multiple comparisons test. Letters denoted significance between different groups at same timepoint; Asterisk denoted significance within same group between different timepoints. D) E) F) Principle-coordinate analysis (PCoA) plots based on Bray-Curtis dissimilarity with ASVs abundance at baseline and 12 hours from donor 1, 2, 3. G) H) I) Relative abundance of ASVs without EAS system from donor 1, 2, and 3.

To analyze the microbial community change in response to oxygen and elemental iron in the *in vitro* fermentation, a total of 72 samples were subjected to 16S rRNA gene v4 sequencing and 1155 reliable ASVs were identified after downsizing to 16,000 reads/sample. Alpha diversity indicates the diversity of gut bacteria community within each sample, which was calculated by Shannon indices (Fig 2A–2C). No significant difference was found in Shannon index at baseline in the samples from all three donors. However, after 12 hours of *in vitro* fermentation, Shannon indices were significantly lower in the oxygen groups compared to the control groups in all three donors. No significant difference was shown between control groups and iron groups in all three donors, revealing that elemental iron did not affect alpha diversity in microbial communities in the absence of oxygen. Interestingly, when we considered the effect of iron on the oxygen-containing groups, it showed donor-dependent variations. The effect of oxygen on alpha diversity was fully reversed by iron treatment in donor 1, showing no significant difference between the control group and the oxygen-iron group. In donor 2 and donor 3, Shannon indices were significantly higher in oxygen-iron groups than in oxygen groups, but still significantly lower than the control groups.

Overall bacteria community shift was visualized by Principle-coordinate analysis plots based on Bray-Curtis dissimilarity in the three donors (Fig 2D–2F). Coinciding with alpha diversities, the control group and the iron group were clustered together at 12 hours in all the three donors, indicating that iron treatment itself did not change the microbiota significantly. In donor 1, the oxygen group shifted along PC2 and separated from the control group, while the oxygen-iron group was close to the control group, which revealed that iron treatment reversed the change induced by oxygen in donor 1. However, the control and the iron groups were clustered together while the oxygen and the oxygen-iron groups clustered and moved along PC2 in donor 2. This suggested that oxygen played the main role in the shift of microbiota structures, while iron did not reverse the oxygen effect in this donor. Meanwhile, the oxygen group was separated from control and iron group along PC2 in donor 3, and oxygen-iron group shifted back along PC2 towards the control group, suggesting that iron treatment partially reversed the oxygen-induced disruption of the gut microbiota. Interestingly, the inter-individual variation of beta diversities matched the pattern of oxidized iron content in three donors, with donor 2 showing the least oxidized iron content as well as no significant protective effect against oxygen, which further confirmed that elemental iron protected gut microbiota through the scavenging of oxygen in the fermentation system.

We hypothesized that elemental iron protects the gut bacteria which lack a defense system against oxygen and reactive oxygen species (ROS). Bacteria without a functional enzymatic antioxidant system (EAS) cannot survive oxygen and are therefore obligate anaerobes. We used PICRUSt2 to predict the presence/absence of the EAS system of 1155 reliable ASVs. ASVs with at least one antioxidant enzyme for superoxide detoxification and one enzyme for hydrogen peroxide decomposition were considered to have functional EAS. We found that there was no significant difference between the control and the iron group in the relative abundance of ASVs without EAS in all three donors. Meanwhile, addition of oxygen significantly reduced the relative abundance of ASVs without EAS in all three donors, indicating oxygen inhibited the growth of ASVs lacking functional EAS. Adding elemental iron in oxygen groups reversed the change in relative abundance of ASVs without EAS in donor 1 and donor 3, but not in donor 2, which coincides with the oxidized iron content and beta diversity data.

### CAG analysis indicates that elemental iron partially reversed oxygen-driven dysbiotic changes in the gut microbiota structure

**Fig 3:**
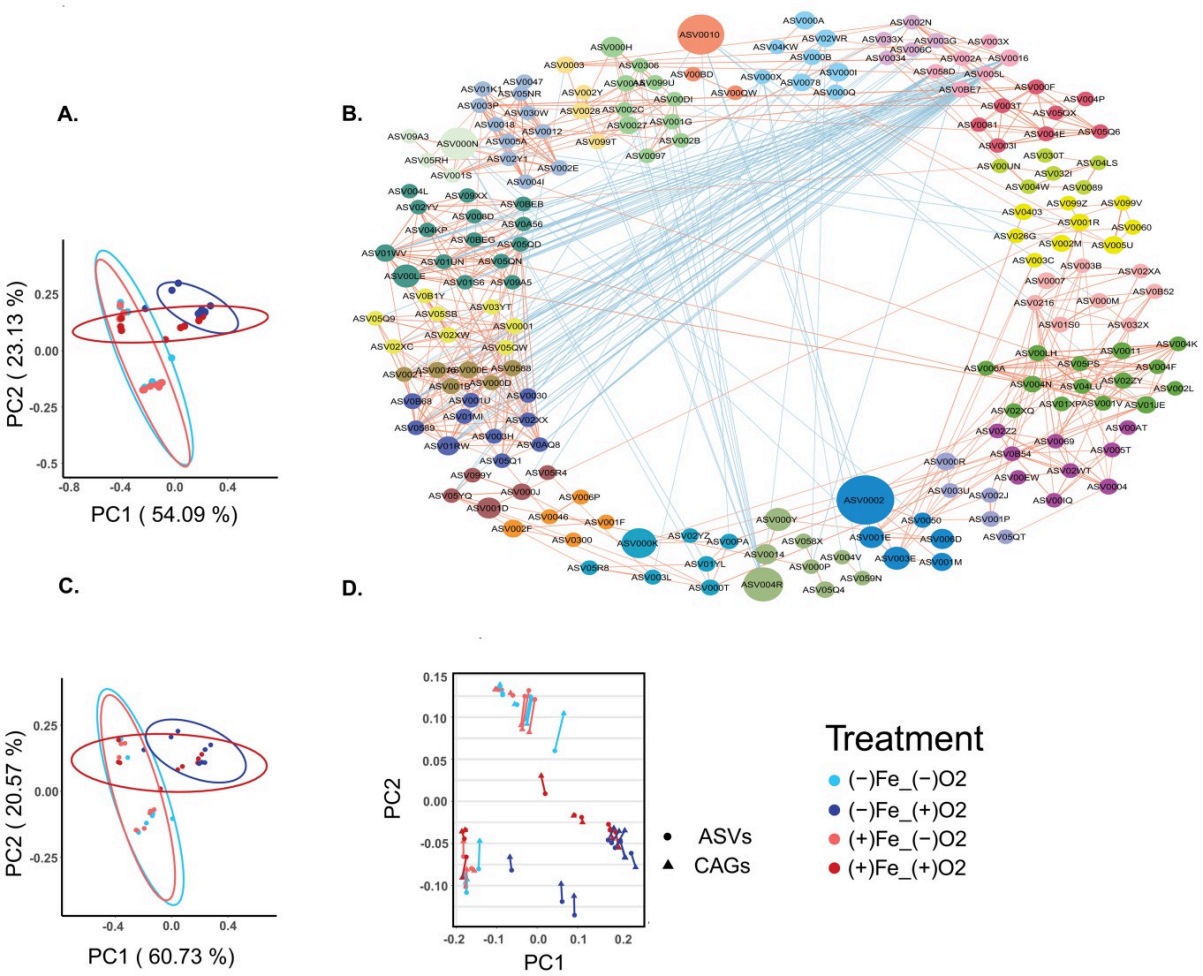
CAGs depicted the gut microbiota structures and indicated that elemental iron partially reversed the dysbiosis caused by oxygen. A) aPCoA plot of gut microbiota (based on Bray Curtis metrics) with ASVs after 12 hours of *in vitro* fermentation. Subject effect adjusted. B) Network plots shows correlations between dominant ASVs within each CAGs. Line width indicates correlations magnitude. Red lines indicate positive correlation and blue lines indicate negative correlation. Correlation > 0.6, p < 0.05 are shown. C) aPCoA plot of gut microbiota with ASVs after 24 hours of *in vitro* fermentation. Subject effect adjusted. D) Procrustes analysis shows CAGs and ASVs with significant correlations.

Since the inter-individual variations among the three donors were significant, we used adjusted Principle-coordinate analysis (aPCoA) to adjust the donor effect. Fig 3A shows the aPCoA plot based on Bray-Curtis dissimilarity with ASV abundance after adjusting subject effect. The control and the iron group were clustered together and showed no significance difference (Stratified PERMANOVA, p = 0.143). The oxygen group was significantly different from the control group, (Stratified PERMANOVA, p = 0.0015). The oxygen-iron group was significantly different from the oxygen group (Stratified PERMANOVA, p = 0.0015) and from the control group (Stratified PERMANOVA, p = 0.0035).

A guild-based analysis was conducted to identify the key bacteria which responded to oxygen and the iron treatment. Within the gut microbiota, bacteria form complex interactions with each other and potentially build up functional groups. Instead of grouping based on taxonomy, bacteria from different taxonomic groups may function as a guild. Members in the same guild exploit the resources in a similar way and show co-abundance behavior. Bacteria that showed co-abundance patterns were assembled to be analyzed as Co-Abundance Groups (CAGs). Instead of taxonomy based analysis, Co-abundance groups revealed the interactions between the bacteria within the microbial community. Out of 1155 ASVs, 215 prevalent ASVs were selected which were shared by more than 30% of samples and accounted for >95% of total abundance, and sequentially clustered into 32 CAGs by repeated measurement function and PERMANOVA analysis. The dynamic network of the CAGs is shown in Fig 3B.

An aPCoA plot based on Bray-Curtis dissimilarity calculated with the abundance of the 32 CAGs (Fig 3C) showed the similar pattern compared to the aPCoA plot with the abundance of the 1155 ASVs (Fig 3A). PC1 accounted for 60.73% of total variation and PC2 explained 20.57%, with significant separation of the oxygen-iron group from the control group (Stratified PERMANOVA, p = 0.0035) and from the oxygen group (Stratified PERMANOVA, p = 0.0019). The similarity of the aPCoA plots between CAGs and ASVs was further confirmed by Procrustes analysis. Procrustes analysis is a statistical analysis used to describe the similarity of shapes. Fig 3D revealed a significant correlation between Fig 3B and 3C (Correlation = 0.98, P = 0.001). The significant similarity indicated that guild-based co-abundance analysis can well capture the structural alterations of gut microbiota induced by oxygen and elemental iron. Co-abundance analysis reduced the high dimensionality in the microbiome without losing ecologically important data. Thus, we used CAGs for further data analysis.

### Unique CAGs were promoted in response to oxygen and elemental iron

**Fig 4:**
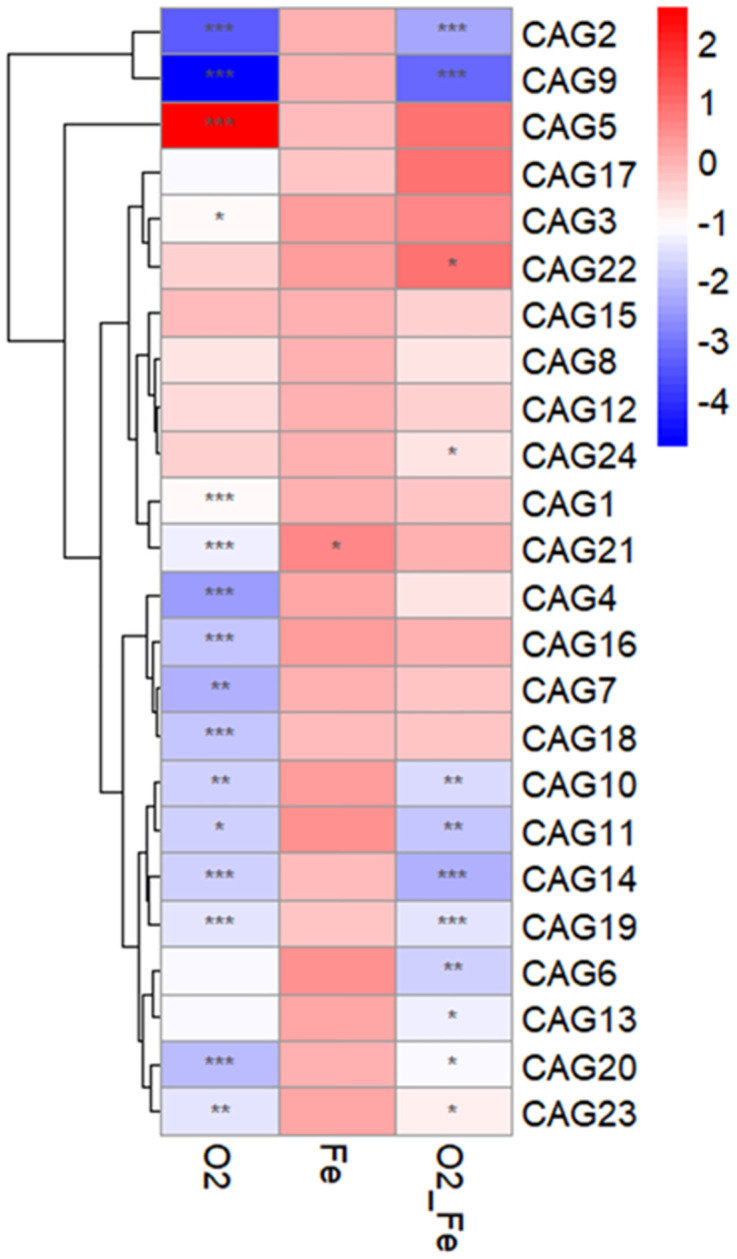
CAGs responding to oxygen and elemental iron after 12 hours of *in vitro* fermentation. Control group at 12 hours as the reference group. MaAslin2 was applied to determine the association. Subject effect was set as random effect.

CAGs responding to oxygen and elemental iron were identified by using MaAslin2, a tool to determine the multivariable association between microbial omics data and the covariates such as host phenotypes or metabolites. The associations between CAGs and treatments (Fig 4) were identified at 12 hours with the control group as reference. When oxygen was introduced into the system, CAG5 showed significantly positive correlation with oxygen, but no significant correlation in oxygen-iron group, indicating that CAG5 may be promoted by oxygen and suppressed by iron. CAG22, on the other hand, showed no significant correlation with the oxygen group or the Iron group, but significantly positively correlated to the oxygen-iron group, indicating oxygen may not inhibit the growth of this CAG but by removing oxygen with iron it may grow better. It was observed that CAG21, CAG1, CAG4, CAG16, CAG7, CAG18 were negatively correlated with oxygen but showed a trend of positive correlation with the oxygen- iron group, suggesting that these CAGs may be inhibited by oxygen and this effect may be reversed by iron treatment.

**Fig 5:**
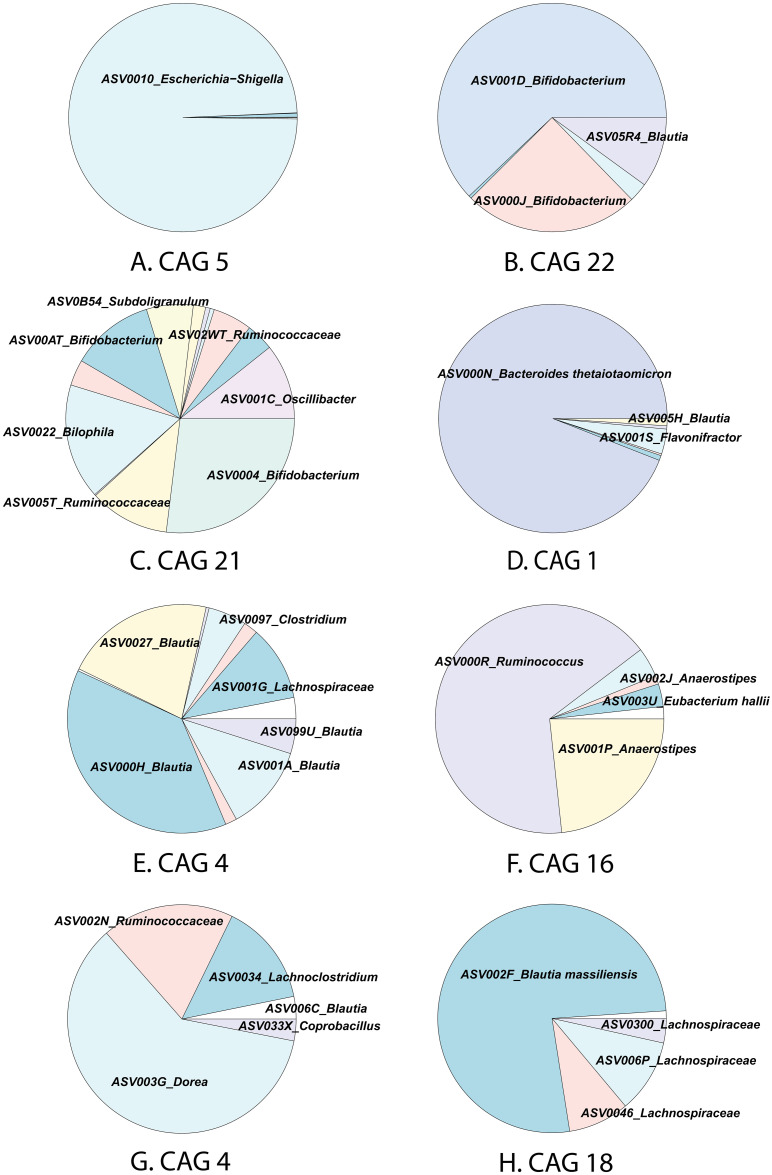
ASV composition of key CAGs at 12 hours of fermentation.

When analyzing the ASVs members in the key CAGs, we found that CAG5 was dominated by *Escherichia Shigella* (Fig 5A), which is a facultative anaerobic bacterium and potentially detrimental. CAG 22 contained two strains of *Bifidobacterium*, *Blautia*, *and Lanchnospiraceae* (Fig 5B), which suggested that these bacteria may tolerate oxygen but grow well when iron scavenged oxygen. CAG 21 was composed of several *Bifidobacterium*, *Bilophila*, *Ruminococcaceae*, *Oscillibacter*, and *Subdoligranulum* (Fig 5C). CAG 1 contains dominantly *Bacteroides thetaiotaomicron*, along with *Blautia*, *Flavonifractor*, and *Fournlerella*.(Fig 5D). CAG 4 was composed of several *Blautia* and *Lachnospiraceae* while CAG 16 was dominated by *Ruminococcus*, *Anaerostipes*, and *Eubacterium halli*. (Fig 5E and 5F). CAG 7 was composed of *Dorea*, *Ruminococcaceae*, *Blautia*, *Coprobacillus*, and *Lachnoclostridum* and CAG 18 contained *Blautia massiliensis* and several sequences of *Lachnospiraceae* (Fig 4G and 4H). Further characterization of the ASVs is needed to understand the interactions between the key CAGs.

### Elemental iron and oxygen induced SCFA change in the gut microbiota with inter-individual variations

Short-chain fatty acids were produced by gut microbiota and serve as important biomolecules to regulate host immunity, maintain epithelial integrity, and inhibit the growth of detrimental bacteria. Acetic acid, propionic acid and butyric acids are the three major SCFA produced in the gut. S1 Fig showed the SCFA incremental change at 12 hours of fermentation compared to the baseline in three donors. Oxygen lowered the production of all three SCFAs compared with control group, which was consistent in all three donors (S1A–S1I Fig). Butyric acid concentration was even lower than the baseline in oxygen group in donor 1 and donor 2 (S1C & S1F Fig). Iron group did not show significant difference in SCFA production compared to control group in donor 1 but showed significant reduced production in donor 2 and 3.

When oxygen was added with iron, SCFA production showed no significant difference from control group in donor 1 (S1A–S1C Fig). However, the production of SCFA was significantly lower than control group and showed no significant difference from oxygen group in donor 2 and donor 3 (S1D–S1I Fig).

The CAGs corresponding to SCFA production were shown in S2 Fig. Most of the CAGs showed negative correlation with the production of SCFAs, possibly due to the high protein content and low fiber content in the growth media. However, CAG 9 and CAG 2 are positively correlated with the production of major SCFA. CAG 9 contains several strains of *Ruminococcaceae*, *Eubacteria Ramulus*, *Blautia*, and *Coprococcus*. CAG 2 is dominated with strains of *Lachnospiraceae* and *Lachnoclostridum*, along with *Alistipes* and *Holdemanella*. Interestingly, CAG 9 and CAG 2 were both repressed by oxygen and the effect was not fully reversed by the addition of elemental iron. This suggested that SCFA producers may be more sensitive to oxygen and hard to recover by elemental iron alone.

## Discussion

In this study we showed that elemental iron reversed the oxygen-induced dysbiosis of human gut microbiota, potentially by scavenging oxygen.

Studies have shown that a high abundance of facultative anaerobic bacteria and low abundance of obligate anaerobes were identified in patients with various diseases such as chronic metabolic syndrome, inflammatory bowel diseases, and HIV [38, 39]. The shift from obligate to facultative anaerobes in the diseased patients may be associated with the disruption of the hypoxic environment in the lumen, which was proposed as the “oxygen hypothesis” [40, 41]. The leakage of oxygen through the epithelial cells enables the buildup of oxygenation along the lumen and provides an ecological advantage to facultative anaerobes which outcompete the obligate anaerobes. On the premise of this hypothesis, we designed this study to test the protective effect of elemental iron against oxygen-induced dysbiosis using the *in vitro* batch fermentation model. By injecting oxygen into the sealed oxygen-free tubes, a microaerobic atmosphere was created in the system and mimicked the oxygenation in the gut. Without considering the host-microbiome interactions, this *in vitro* model provides a simple and ecologically meaningful method to investigate causality between the environment-microbiota interactions.

Fecal fermentation was performed in eutrophic BCM medium (versus oligotrophic PBS medium). This medium is used to simulate substances that reach the colon, when a host is subjected to a high fat high protein diet [22, 42]. Recent studies indicate that high protein high-fat diets can reduce gut microbial diversity [43]. Moreover, food digestion through proteolytic catabolic pathway not only alters SCFA formation, but also leads to other potentially toxic co-metabolites [44–47]. In accordance with this, excessive consumption of animal protein was associated with an increased risk of developing Crohn’s disease (CD) [48]. We modified BCM by mucin supplementation in order to promote growth of *A*. *muciniphila*, potentially beneficial gut bacterium, associated with favorable metabolic outcomes and removing reducing agent cysteine hydrochloride (to avoid changes in oxygen content after oxygen injection and competing for oxygen with iron) [23, 24].

During *in vitro* fermentation, iron oxidation patterns were measured at 0 and 12 h. Initially, we also determined oxidized iron content (for donors 1 and 2) at 2, 24 and 48 h. There was no statistically significant difference in oxidized iron content in the samples exposed to oxygen for 12 h compared to 2 and 24 h in both eutrophic BCM and oligotrophic PBS media. Therefore, we consider the results for 12 h representative for further cultivation points.

Notably, inter-individual variation of iron oxidation was observed in the three donors correlating with the pattern of the oxygen-disrupted microbiota restoration by iron treatment. Thus, in BCM the percentage of oxidized iron was highest in donor 1 and lowest in donor 2. This indicates that the more oxygen was scavenged by elemental iron, the less facultative anaerobes the oxygen could promote. We used the same culturing condition for all the three donors. For donor 2, we collected the fecal samples twice and repeated the *in vitro* fermentation in two separate days. Both experiments resulted in low oxidized iron content. This suggests that the oxygen-scavenging efficiency of iron may be influenced by the initial gut microbiota composition.

We also measured iron oxidation patterns in oligotrophic PBS medium in conditions that otherwise were similar to those during fermentation in BCM. We used PBS to simulate normal / calorie-restricted diet, in which available nutrients are absorbed primarily while passing through the upper parts of the GI tract [22]. Our findings demonstrate that oxidized iron content in the BCM samples is significantly lower than in the PBS samples for donors 1 and 2, while similar results were obtained for donor 3 on both media. Notably, donor 2 samples, cultured in PBS, contained less oxidized iron than the corresponding donor 1 samples. This suggests that growth medium as well as donor microbiota may affect oxygen neutralization level (as measured by oxidized iron content). Thus, complementing iron supplementation with other treatments, e.g., specific diet (e.g., less nutrient rich) might improve gut microbiota restoration by iron. In addition, these findings corroborate the importance of trophic status of a diet in development and subsequent treatment of microbial dysbiosis [49, 50].

Notably, the inter-individual variation of oxidized iron content matched alpha and beta diversities pattern in three donors. Thus, alpha diversity was significantly lower in the oxygen groups compared to the control groups in all three donors, indicating that oxygen perturbed the microbiota and lowered the diversity of the bacterial community, while elemental iron did not affect alpha diversity in microbial communities in the absence of oxygen. In the oxygen-containing samples elemental iron partially reversed the microbiota disruption in donor 2 and donor 3, while showing full restoration in donor 1 in terms of alpha diversity.

Coinciding with alpha diversities, beta diversity metrics indicated that iron treatment itself did not change the microbiota significantly. In donor 1 iron treatment reversed the change induced by oxygen. However, in donor 2 oxygen played the main role in the shift of microbiota structures, while iron did not reverse the oxygen effect in this donor. In donor 3, iron treatment partially reversed the oxygen-induced changes to the gut.

Analysis of ASVs lacking functional EAS, correlated with beta diversity patterns. Thus, oxygen inhibited the growth of putative anaerobic bacteria in all three donors. Elemental iron in oxygen groups reversed the changes in the relative abundance of ASVs without EAS in donor 1 and 3, but not in donor 2. Matching patterns of the performed analyses in three donors further confirm that elemental iron could protect gut microbiota through the scavenging of oxygen in the fermentation system.

Furthermore, we analyzed the effect of oxygen and iron administration on ecologically / functionally connected groups of bacteria (guilds) [51]. We found that CAG 5 was significantly promoted by oxygen. *Escherichia-Shigella* was the dominant ASV in CAG 5 and belonged to *Enterobacteriaceae*, a facultative anaerobe [52–56]. *Escherichia* was identified as significantly increased in Crohn’s Disease (CD) patients compared to healthy controls, indicating that the onset of CD was associated with these proinflammatory bacteria [52]. Another study had shown that *Escherichia* was remarkably enriched in Chinese patients with ulcerative colitis [53]. *Escherichia/Shigella* were important distinguishing genera in polycystic ovary syndrome (PCOS) [54]. Notably, expansion of *Enterobacteriaceae* could be correlated with the increase in nitrate and oxygen levels in the inflamed gut described in IBD patients [55]. After adding elemental iron into the oxygenated fermentation system, *Escherichia-Shigella* was not significantly promoted in oxygen-iron group, indicating that elemental iron may have reduced the available oxygen in the system.

When oxygen and elemental iron were both present in the system, *Bifidobacterium* ASV001D and ASV000J as well as *Blautia* 05R4 were significantly promoted. *Bifidobacterium* was reported to ameliorate visceral fat accumulation in a mouse model of metabolic syndrome [57]. *Blautia* was also studied as a potential probiotic and negatively associated with the inflammation and obesity [58]. In the meantime, bacteria which were repressed by oxygen, such as *Ruminococcus*, *Bacteroides thetaiotaomicron*, *Eubacterium hallii and Lanchnospiraceae* were rescued by elemental iron. *Bacteroides thetaiotaomicron* is an obligate anaerobic bacterium and also an opportunistic pathogen [59, 60]. However, it was also reported to promote the integrity of the mucosal barrier, regulate the immune system, and reverse obesity and insulin resistance [61, 62]. *Eubacterium hallii* contributed to the formation of SCFAs, which are an energy source for the colonocytes and may mitigate inflammation [26]. *Ruminococcus and Lanchnospiraceae* are reported to be obligate anaerobes while the specific strains promoted by iron need further characterization [63, 64].

Regarding SCFA production, the iron group did not show significant difference compared to control group in donor 1, but showed significantly reduced production in donor 2 and 3. However, the diversity and the structure of microbial communities showed no significant change when elemental iron alone was introduced in the system. As less elemental iron was oxidized in donor 2 and donor 3 samples compared to donor 1 samples, it is possible that reduced iron may interact with SCFA or molecular precursors in a way that oxidized iron does not, resulting in this discrepancy between donor 1 and donors 2 and 3. It is worth noting that potentially opportunistic bacteria can also produce SCFA together with potentially toxic co-metabolites [44–47]. Interestingly, fecal microbiota of donor 2 generated the highest SCFAs in the control group after 12 hours. The relationship between SCFA production and oxygen-scavenging efficiency of microbiota needs to be studied in the future. The excess administration of heme iron was reported to be associated with the aggravation of colon cancer [65]. Most pathogenic bacteria have heightened iron acquisition mechanisms and therefore tend to outcompete protective bacteria for free iron [56, 65]. Depending upon the route of administration to colorectal cancer patients, iron therapy has the potential to contribute to a procarciongenic microbiota [56, 65]. Currently, this has only been assessed in murine studies, with human trials being necessary to unravel the potential microbial outcomes of iron therapy [65]. However, whether elemental iron showed a similar effect remains elusive.

In conclusion, we found that the extent of oxygen scavenging effect of elemental iron is donor dependent. Four key CAGs were identified as potential guilds responsive to oxygen and elemental iron treatments. Future research is warranted to investigate the cause of the discrepancy in degree of responsiveness of donor microbiota to elemental iron treatment, and whether factors such as donor diet play a role in these differences.

## Supporting information

S1 Fig Change in short chain fatty acid concentrations following 12h fermentation. Difference in acetic acid, propionic acid, and butyric acid contentrations at 12h for A-C) donor 1, D-F) donor 2, and G-I) donor 3 relative to 0h concentrations.
(TIF)

S2 Fig SCFAs corresponded to CAGs at 12 hours of *in vitro* fermentation. MaAslin2 was applied to determine the association. Subject effect was set as random effect.
(TIF)

S1 Data (XLSX)

S2 Data (XLSX)

S3 Data (XLSX)

S4 Data (XLSX)

S5 Data (XLSX)
