# Supplementary figures and images for: Elemental iron protects gut microbiota against oxygen-induced dysbiosis
Source: PLoS One. 2024 Feb 27;19(2):e0298592. doi: 10.1371/journal.pone.0298592 (PMC10898728; doi:10.1371/journal.pone.0298592)

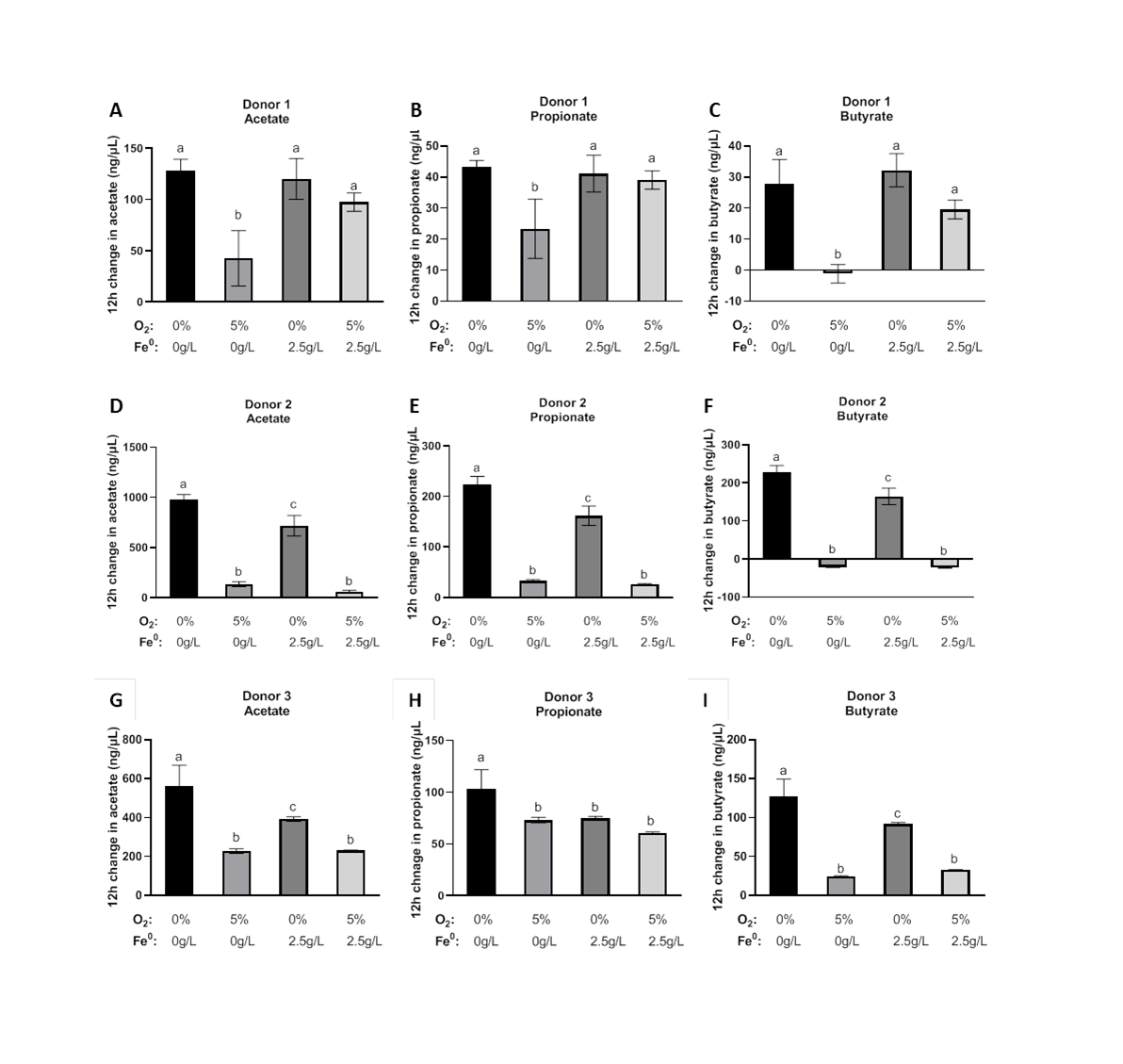

Supplement: S1 Fig — Difference in acetic acid, propionic acid, and butyric acid contentrations at 12h for A-C) donor 1, D-F) donor 2, and G-I) donor 3 relative to 0h concentrations. (TIF) [file pone.0298592.s001.tif]

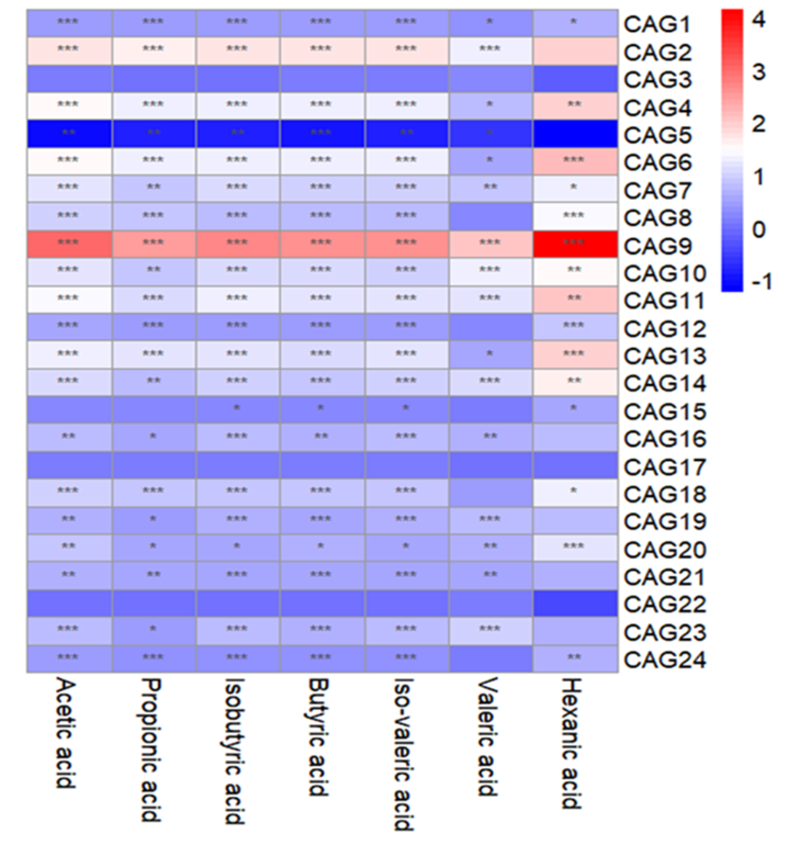

Supplement: S2 Fig — MaAslin2 was applied to determine the association. Subject effect was set as random effect. (TIF) [file pone.0298592.s002.tif]
